# Relationship between Burnout, Quality of Life, and Work Ability Index—Directions in Prevention

**DOI:** 10.1100/tsw.2010.83

**Published:** 2010-05-04

**Authors:** Mirjana Arandjelovic, Maja Nikolic, Slavisa Stamenkovic

**Affiliations:** ^1^ Institute of Workers' Health Protection, Faculty of Medicine, University of Niš, Serbia; ^2^ Institute of Public Health, Faculty of Medicine, University of Niš, Serbia; ^3^ Department of Biology and Ecology, Faculty of Natural Sciences and Mathematics, University of Niš, Serbia

**Keywords:** burnout, quality of life, work ability index, food manufacturing, prevention, salutogenesis

## Abstract

The aim of our study was (1) to test the possibilities of standardized questionnaires for burnout, quality of life, and work ability in Serbia by investigating interactions of these phenomena in food manufacturing workers in Serbia; and (2) to determine possible preventive measures. The study enrolled 489 food manufacturing workers in the region of Niš (Serbia) during the period from January 2008 to February 2009. We included three standardized questionnaires: for burnout (CBI), quality of life (ComQoL-A_5_), and the work ability index (WAI) in the Serbian language. The results of our study indicate high scores in personal (60.0) and work burnout (67.9), lower scores for objective (66.2%SM) and subjective quality of life in enrolled subjects (69.2%SM), and an excellent work ability index in most workers (65.8%). The questionnaires tested are reliable instruments in the Serbian region. Burnout, quality of life, and work ability are significantly interrelated categories in food manufacturing workers. There is a high degree of work burnout that has not yet been accompanied with significant impairment of quality of living and work ability in exposed workers. That is why a salutogenic approach in the prevention of this phenomenon, by health-promotion programs in the workplace, would be the method of choice for burnout improvement.

## INTRODUCTION

Serbia is one of the countries in transition. Recent data indicate that 35% of its population suffers from post-traumatic stress disorder due to the civil war in the surrounding areas, and a collapse of the former state. The mental health care system has been seriously affected by these events, so overall quality of services is not satisfactory. An additional problem is the economic and financial crisis that also compromises the health care system of the country. There are many limitations regarding the financial assets, legislatives, and long-term strategies. The level of consciousness among the decision-making authorities and the people in the country regarding health care and the working population is also not satisfactory.

Deteriorating moral values, great unemployment, globalization, job uncertainty, an increase in the number of people working in small and medium-sized companies, and inevitable continuous education are additional stressors. At the same time, attitudes towards work have changed. The job is not merely a source of income, but provides social contacts and plays an important role in self-esteem and identification. Work plays a central role in people's physical and psychological well-being[1,2]. Occupational health care workers identified complaints from other workers on longer work hours, exhaustion, lack of free time, sleep disorders, work-associated withdrawal behavior, and anxiety. Many of them manifested burnout syndrome. A commonly discussed source of burnout is overload: job demands exceeding human limits. People have to do too much in too little time with too few resources. Increased workload has a consistent relationship with burnout, especially in combination with the exhaustion dimension[3,4].

Serbia submitted its application to join the EU and certain laws would become applicable in the field of occupational issues, leading to better work organization. Last year, the law on work safety started to be implemented, and employers became obligated to assess work risk factors and perform preventive measures. The identified increased professional stress, as a risk factor, appears in the Act on Risk Assessment of many companies, but the possibilities for acting according to it are a problem. First of all, there are no valid questionnaires in the Serbian language regarding evaluation of such risks. Preventive health promotion is not obligatory for employers.

Health promotion is a cultural, social, environmental, economic, and political process. Health promotion and disease prevention can perhaps, in a sense, be seen as two sides of the same coin. Human rights are fundamental to health promotion and a basis for equity, empowerment, and engagement[5]. However, in Serbia, the Labor Law that promotes health in the workplace has not yet been properly implemented. This is a problem because basic occupational health protection is not sufficient for overall health protection. The economy of the country is based on exchange of goods, not on capital transfer, which cannot be helpful for the health-protection system of the country. In this situation, the capabilities of the individual, the group, and the society to manage the changes become crucial. The way people are able to perceive structures, create coherence, and keep everything together has a central impact on health[6]. The policy of the state and legislature regarding this problem has a huge importance.

Each transition is a sensitive period that makes us vulnerable to change. On the other hand, it gives us possibilities to mobilize our resources to make the transition manageable and to acquire new life experiences. Last year, the authors of this study realized the project No 21016: "Introduction and development of the coherent engagement of all economic branches and their employees in promotion of health, ethics and quality of living as a condition for their development". As a result, the first regional center for health promotion in the workplace was opened in Niš (Serbia), and the International Code of Ethics for Occupational Health Professionals in the Serbian language, issued by the International Commission on Occupational Health (2002), was adopted. Upon completion of the study, the authors faced some other questions: How do we manage the consequences of accumulated stress in workers in Serbia? What are other experiences regarding stress prevention in the workplace? Can the experience of other researchers and their instruments be applicable to our country? How do we direct the prevention and how do we come up with an organization for the newly opened center for health promotion in the workplace?

Mental health protection for workers is an urgent need, as indicated by the European Network for Workplace Health Promotion – ENWHP: "Work. In tune with life. Move Europe – Step by step". Mental health is central to the human, social, and economic capital of society. The mental health intervention programs should involve actions to reduce or eliminate occupational stressors, and to create individual, social, and environmental conditions that enable optimal overall psychological development[5,6].

The aim of this study was (1) to test the possibilities for application of the questionnaires for burnout, quality of living, and work ability translated into the Serbian language, by monitoring interactions of these phenomena in food manufacturing workers; and (2) to review other authors' studies in order to find possible preventive measures and to draw the attention of the state and other researchers to the topics of this study.

## METHODS AND MATERIALS

The study was conducted among 514 food manufacturing workers in the Niš region during the period from January 2008 to February 2009 at the Institute for Health Protection. Out of a total number of examined workers, 489 (95%) adequately answered all the questions, while 25 (5%) of the questionnaires could not be used for the analysis. The mean time for completing the questionnaires was 40 min. The participation in the study was voluntary and anonymous. All the subjects were given a special survey that included questionnaires on burnout, the Copenhagen Burnout Inventory (CBI); quality of life, the Comprehensive Quality of Life Scale – Adult, 5th edition (ComQoL-$A_5$); and the Work Ability Index (WAI).

### Questionnaires

- The **Copenhagen Burnout Inventory (CBI)** — A 19-item multiple-choice questionnaire (five items for an answer) measuring three burnout subdimensions: the personal burnout scale (six items), work-related burnout (seven items), and client-related burnout (six items). Some scales can be used independently in accordance with the examined population. Items are rated on the Likert scale. Values of all the scales range from 0 to 100 points. High scores indicate high burnout. Mean scores of all the scales indicate burnout is low if it is less than 50 points (<50), and high if it is over 50 points (50+). In our study, we used personal burnout and work-related burnout. The third subscale on client-related burnout was not used since the subjects of the study were manufacturing workers, not service industry workers[4,7].
- The **Comprehensive Quality of Life Scale (ComQol)** — In this study, we used the 5th edition of the ComQol scale on adults (ComQoL-$A_5$) that was created in 1997. This instrument is based on the following statements: quality of life comprises objective and subjective components, and both components comprise seven domains: material well-being, health, productivity, family and friends relations, feeling of security, feeling of belonging to a community, and emotional well-being. So, in the questionnaire, each of the seven domains is treated with one question in three sections: present situation, importance, and satisfaction with each domain. The items in the questionnaire were graded according the Likert scale, ranging from 0 to 100 points. According to given forms, obtained values are transformed into scores for objective and subjective quality of living as a percentage of maximum scale value (%SM). The scores from the first section are the scores of the objective quality of life. Multiplying scores for importance and satisfaction represent the subjective component of quality of life. According to the research published so far, favorable standards are the values of 70–80%SM[8,9].
- The **Work Ability Index (WAI)** — The WAI developed by the Finnish Institute of Occupational Health is a questionnaire-based method assessing perceived work ability. The WAI score was calculated as the sum of seven items: current work ability compared with the lifetime best, work ability in relation to the demands of the job, number of current diseases diagnosed by a physician, estimated work impairment due to diseases, sick leave during the past 12 months, personal prognosis of work ability 2 years from now, and mental resources. The WAI score ranged from 7 to 49 points. Higher scores indicate better work ability. The WAI is considered *poor* in the range from 7 to 27, *moderate* in the range from 28 to 36, *good* in the range from 37 to 43, and *excellent* in the range from 44 to 49. Subjects at 36 points or below were classified as having *low work ability*. Subjects at 37 points or above were classified as having *satisfying work ability*[10]

## Statistical Analysis

A tool for assessing the reliability of scales for burnout, quality of living, and work ability was done by the Cronbach $\alpha$ coefficient that determines the internal consistency of scale items. The lower limit of an acceptable reliability coefficient of the scales was 0.60, and the lower limit of high reliability was 0.70[11].

Regression analysis was applied to assess factors important for quality of living and work ability. It was based on a univariate regression model, then a multivariate model was introduced by backward stepwise method, excluding the factors with no significant impact on dependant variables. Sex in the models was defined as a changeable value, and all the other factors were defined as continuous numeric values. Coefficients of linear regression (B) and probability of error in their assessment (p) were calculated and presented. Values of coefficients represent change in scores in dependant variables caused by value changes in independent ones for one measure unit. As for sex, that is independently changeable; regression coefficient represents change of scores in dependant variables in women compared to men.

Since objective and subjective quality of living are multidimensional, multivariate analysis of variance (MANOVA) was conducted in order to test effects of personal burnout, burnout at work, age, and sex. Objective and subjective quality of life domains (seven domains) were introduced as dependent variables in MANOVA models. The influence of individual as well as interacting important factors on domains was assessed by calculating F values and probability of error (p).

In all the analyses, the limit of statistical significance was evaluation error of 0.05 or 5%. Calculations were done in the S-PLUS program, version 2000.

## RESULTS

The study enrolled 489 food manufacturing workers in Niš. The mean age was 45 (25–57) among 262 males (53.6%) and 227 females (46.4%). Personal burnout mean score was 60.0 points, while burnout at work was determined by the mean value of 67.9. Total mean score for objective quality of living in the respondents of our study was 66.2% out of maximum values scale and for subjective quality of living, it was 69.2% out of the same scale. In certain domains of objective quality of living, the highest value of the subscore was in the health domain (91.7%SM) and in productivity (83.3%SM), and the lowest was in the domain of emotional well-being (50.0%SM). In the domain of subjective assessment of quality of life, the highest value of the subscore was in the health (56.1%SM) and intimacy (56.1%SM) domain, and the lowest in material well-being (52.6%SM). The WAI was excellent in 65.8%, good in 24.5%, and moderate in 9.6% of respondents (see Table 1).

Values of Cronbach $\alpha$ coefficient indicate high reliability of scales in measuring personal burnout (0.87), burnout at work (0.86), and subjective quality of life (0.83), while internal consistency of scale factors for determination of objective quality of life (0.66) and work ability index (0.68) was slightly lower, but within accepted reliability (see Table 2).

Regression analysis confirmed the high statistically significant impact of increase of personal burnout and burnout at work on decrease of values of the total score of objective and subjective quality of life. Out of some domains of objective quality of life, with adjustment to effects of sex and age, increased personal burnout is associated to statistically significant deterioration of health (B = –0.022), productivity (B = –0.017), intimacy (B = –0.036), and safety (B = –0.052). A significant influence of personal burnout on material and emotional well-being, as well as on social status, is not confirmed. Increased burnout at work is associated with a statistically significant increase of material well-being (B = 0.017), but also with a significant decrease in productivity (B = –0.022), intimacy (B = –0.022), and safety (B = –0.040). Analysis of the connection with some domains of subjective quality of life, controlling the influence of age and sex, indicated that increased personal burnout is accompanied by a statistically significant decrease of material well-being (B = –0.031), health (B = –0.145), intimacy (B = –0.058), and social status (B = –0.066), while increased burnout at work is associated with significant deterioration and

**TABLE 1**
**Characteristics of Respondents: Age, Sex, Burnout, Quality of Life and Work Ability Index Scores**

| Characteristics | No. of Respondents | Mean (Minimum–Maximum) | Frequency (%) |
|---|---|---|---|
| Age (years) | 489 | 45.0 (25.0–57.0) | |
| Sex | | | |
| *Men* | 262 | | 53.6 |
| *Women* | 227 | | 46.4 |
| Burnout | | | |
| *Personal burnout* | 489 | 60.0 (8.3–100.0) | |
| *Work burnout* | 489 | 67.9 (10.7–85.7) | |
| Objective quality of life | 489 | 76.6 (58.6–88.8) | |
| % SM | | 66.2 (44.8–80.7) | |
| *Material well-being* | 489 | 11.0 (7.0–13.0) | |
| *% SM* | | 66.7 (33.3–83.3) | |
| *Health* | 489 | 14.0 (5.0–15.0) | |
| *% SM* | | 91.7 (16.7–100.0) | |
| *Productivity* | 489 | 13.0 (8.0–15.0) | |
| *% SM* | | 83.3 (37.5–100.0) | |
| *Intimacy* | 489 | 11.0 (3.0–15.0) | |
| *% SM* | | 66.7 (0.0–100.0) | |
| *Safety* | 489 | 12.0 (3.0–14.0) | |
| *% SM* | | 75.0 (0.0–91.7) | |
| *Social status* | 489 | 7.8 (5.2–15.0) | |
| *% SM* | | 40.0 (18.3–100.0) | |
| *Emotional well-being* | 489 | 9.0 (5.0–13.0) | |
| *% SM* | | 50.0 (16.7–83.3) | |
| Subjective quality of life | 489 | 58.0 (-44.0–96.0) | |
| % SM | | 69.2 (36.1–83.5) | |
| *Material well-being* | 489 | 6.0 (-10.0–12.0) | |
| *% SM* | | 52.6 (43.9–57.9) | |
| *Health* | 489 | 10.0 (-10.0–20.0) | |
| *% SM* | | 56.1 (43.9–64.9) | |
| *Achievements* | 489 | 8.0 (-16.0–16.0) | |
| *% SM* | | 54.4 (38.6–61.4) | |
| *Intimacy* | 489 | 10.0 (-6.0–20.0) | |
| *% SM* | | 56.1 (47.4–64.9 | |
| *Safety* | 489 | 8.0 (-20.0–15.0) | |
| *% SM* | | 54.4 (35.1–60.5) | |
| *Social status* | 489 | 8.0 (-16.0–20.0) | |
| *% SM* | | 54.4 (38.6–64.9) | |
| *Emotional well-being* | 489 | 8.0 (-16.0–15.0) | |
| *% SM* | | 54.4 (38.6–60.5) | |
| Work ability index | 489 | 45.0 (32.0–49.0) | |
| *Excellent (44–49)* | 322 | | 65.8 |
| *Good (37–43)* | 120 | | 24.5 |
| *Moderate (28–36)* | 47 | | 9.6 |
| Low (7–27) | — | | — |

**TABLE 2**
**Reliability Analysis for Measuring Burnout, Quality of Life, and Work Ability Index**

| Scale | No. of Factors | Cronbach α |
|---|---|---|
| Personal burnout | 6 | 0.87 |
| Work burnout | 7 | 0.86 |
| Objective quality of life | 7 | 0.66 |
| Subjective quality of life | 7 | 0.83 |
| Work ability index | 7 | 0.68 |

decrease of health (B = –0.092), intimacy (B = –0.047), safety (B = –0.054), and social status (B = –0.093) (see Table 3). MANOVA indicates highly statistically significant interactive and single effects of personal burnout, burnout at work, age, and sex on the scores of all domains of objective and subjective quality of life (see Table 4). Univariate regression analysis confirmed that increased personal burnout (B = –0.041) and burnout at work (B = –0.029), as well as female sex (B = –1.048) are associated with statistically significant decrease in the work ability index, while an increase in scores of objective (B = 0.224) and subjective (B = 0.049) quality of life is associated with significant increase of work ability index. Multivariate regression analysis distinguishes objective (B = 0.125) and subjective (B = 0.027) quality of life, as well as female sex (B = –0.909) as factors significantly associated with work ability index (see Table 5).

## DISCUSSION

In the framework of modern living conditions and global trend consequences, doctors of occupational medicine among economically active populations are faced with an increased need for prevention of the unfavorable impact of psychosocial factors on workers' health and their work ability. Such a need is especially significant in the countries in economic and health care transition. Although Serbia is one of these countries, the mentioned phenomena were scarcely the subject of interest, so there are no special instruments for this kind of investigation. On the other hand, there have been many discussions, studies, and projects in the world in recent years that were aimed at finding appropriate questionnaires for burnout and quality of life of workers of different professions[4,12,13]. The choice of questionnaires in this study was made due to the possibility to use their single scales separately. Testing of the questionnaires that we translated and used in this study[7,9,10] indicated the reliability of all the scales, meaning that they can be used in our future investigations, as well as in investigations of other authors in the country.

The results of our study indicate high scores for personal and work burnout, and lower scores for quality of life, in our respondents compared with those that are considered to be the expected standard[8,9], and an excellent work ability index in most workers. Such results could be primarily explained by general characteristics and high demands of the period of time in which the respondents live and work, referring to actual privatisation of the enterprises, fear of losing their jobs, maladjustment, low salaries, etc. that contribute and intensify the already known usual stressors in everyday life of each individual in modern society. Taking into account burnout as a dynamic process, and unfavorable effects of microstressors in a long run without warning, the high work ability index in workers in this study can be a consequence of workers' enthusiasm, as defined in the burnout definition, and as a consequence of fear of losing their job as well and their endeavor to keep it, and that requires a special analysis. We believe that it is especially important in such a situation to make a good choice of methods of intervention programs in prevention, aiming to prevent burnout and all the consequences that accompany it, and at the same time, to maintain an excellent work ability index, otherwise the consequences can be serious. The intervention program in this case depends on the results of provoking factors investigations, although it seems complex.

**TABLE 3**
**Regression Analysis of Personal and Work Burnout Influence on Objective and Subjective Quality of Life**

| Indicators of Quality of Life | Univariate Analysis | | | | | | Multivariate Analysis[a] | | | | | |
|---|---|---|---|---|---|---|---|---|---|---|---|---|
| | B | Standardized B | R Square | 95% CI for B Lower Bound | 95% CI for B Upper Bound | p | B | Standardized B | R Square | 95% CI for B Lower Bound | 95% CI for B Upper Bound | p |
| | | | | | Personal Burnout | | | | | | | |
| Objective quality of life | -0.123 | -0.344 | 0.118 | -0.153 | -0.093 | * | -0.132 | -0.366 | 0.134 | -0.165 | -0.100 | * |
| *Material well-being* | -0.001 | -0.020 | 0.000 | -0.008 | 0.005 | — | 0.001 | 0.007 | 0.008 | -0.006 | 0.008 | — |
| *Health* | -0.022 | -0.234 | 0.055 | -0.030 | -0.014 | * | -0.022 | -0.232 | 0.071 | -0.031 | -0.013 | * |
| *Productivity* | -0.007 | -0.086 | 0.007 | -0.014 | 0.000 | — | -0.017 | -0.207 | 0.160 | -0.025 | -0.010 | * |
| *Intimacy* | -0.032 | -0.260 | 0.068 | -0.042 | -0.021 | * | -0.036 | -0.298 | 0.131 | -0.047 | -0.025 | * |
| *Safety* | -0.053 | -0.504 | 0.254 | -0.061 | -0.045 | * | -0.052 | -0.494 | 0.240 | -0.061 | -0.044 | * |
| *Social status* | -0.002 | -0.023 | 0.001 | -0.011 | 0.007 | — | -0.005 | -0.051 | 0.101 | -0.015 | 0.004 | — |
| *Emotional well-being* | -0.006 | -0.060 | 0.004 | -0.014 | 0.003 | — | 0.001 | 0.005 | 0.051 | -0.009 | 0.010 | — |
| Subjective quality of life | -0.311 | -0.207 | 0.043 | -0.442 | -0.180 | * | -0.297 | -0.195 | 0.039 | -0.440 | -0.154 | * |
| *Material well-being* | -0.018 | -0.058 | 0.003 | -0.044 | 0.009 | — | -0.031 | -0.099 | 0.017 | -0.060 | -0.001 | *** |
| *Health* | -0.143 | -0.523 | 0.273 | -0.163 | -0.122 | * | -0.145 | -0.526 | 0.265 | -0.168 | -0.123 | * |
| *Productivity* | 0.001 | 0.004 | 0.000 | -0.022 | 0.025 | — | -0.002 | -0.006 | 0.040 | -0.027 | 0.024 | — |
| *Intimacy* | -0.050 | -0.225 | 0.050 | -0.069 | -0.030 | * | -0.058 | -0.257 | 0.085 | -0.078 | -0.037 | * |
| *Safety* | -0.027 | -0.079 | 0.006 | -0.057 | 0.003 | — | -0.014 | -0.040 | 0.018 | -0.046 | 0.018 | — |
| *Social status* | -0.068 | -0.199 | 0.040 | -0.098 | -0.038 | * | -0.066 | -0.196 | 0.045 | -0.096 | -0.036 | * |
| *Emotional well-being* | -0.008 | -0.021 | 0.000 | -0.039 | 0.024 | — | 0.000 | -0.001 | 0.006 | -0.033 | 0.032 | — |
| | | | | | Work Burnout | | | | | | | |
| Objective quality of life | -0.086 | -0.215 | 0.046 | -0.121 | -0.051 | * | -0.078 | -0.195 | 0.047 | -0.114 | -0.042 | * |
| *Material well-being* | 0.013 | 0.160 | 0.025 | 0.006 | 0.020 | * | 0.017 | 0.209 | 0.051 | 0.010 | 0.024 | * |
| *Health* | -0.009 | -0.085 | 0.007 | -0.018 | 0.000 | — | -0.009 | -0.087 | 0.029 | -0.019 | 0.000 | — |
| *Productivity* | -0.018 | -0.195 | 0.038 | -0.026 | -0.010 | * | -0.022 | -0.236 | 0.176 | -0.029 | -0.014 | * |
| *Intimacy* | -0.026 | -0.190 | 0.036 | -0.038 | -0.014 | * | -0.022 | -0.166 | 0.076 | -0.034 | -0.010 | * |
| *Safety* | -0.044 | -0.372 | 0.139 | -0.053 | -0.034 | * | -0.040 | -0.342 | 0.129 | -0.050 | -0.030 | * |
| *Social status* | -0.005 | -0.047 | 0.002 | -0.015 | 0.005 | — | -0.006 | -0.052 | 0.102 | -0.016 | 0.004 | — |
| *Emotional well-being* | 0.002 | 0.022 | 0.000 | -0.007 | 0.012 | — | 0.005 | 0.044 | 0.053 | -0.005 | 0.015 | — |
| Subjective quality of life | -0.378 | -0.226 | 0.051 | -0.524 | -0.233 | * | -0.334 | -0.199 | 0.042 | -0.487 | -0.182 | * |
| *Material well-being* | -0.023 | -0.067 | 0.004 | -0.052 | 0.007 | — | -0.029 | -0.085 | 0.015 | -0.060 | 0.002 | — |
| *Health* | -0.097 | -0.318 | 0.101 | -0.123 | -0.071 | * | -0.092 | -0.302 | 0.098 | -0.119 | -0.065 | * |
| *Productivity* | -0.017 | -0.058 | 0.003 | -0.043 | 0.009 | — | -0.011 | -0.037 | 0.041 | -0.038 | 0.016 | — |
| *Intimacy* | -0.048 | -0.194 | 0.037 | -0.069 | -0.026 | * | -0.047 | -0.189 | 0.060 | -0.069 | -0.025 | * |
| *Safety* | -0.061 | -0.163 | 0.027 | -0.095 | -0.028 | * | -0.054 | -0.145 | 0.037 | -0.088 | -0.020 | ** |
| *Social status* | -0.105 | -0.276 | 0.076 | -0.137 | -0.72 | * | -0.093 | -0.245 | 0.069 | -0.126 | -0.059 | * |
| *Emotional well-being* | -0.028 | -0.071 | 0.005 | -0.063 | 0.007 | — | -0.008 | -0.020 | 0.012 | -0.044 | 0.028 | — |

[a] Adjusted to sex and age.

*****$p < 0.001$, **$p < 0.01$, ***$p < 0.05$.

The phenomenon we investigated in our study is multifactorial and it allows the possibility of different approaches to the analysis. We were interested in the interrelation of burnout that can seriously damage workers' health and the things that are significant for each of them, quality of life, and the ability to meet the demands of everyday work.

The analysis of burnout and subjective quality of life in our study indicates that burnout is accompanied by a statistically significant deterioration of health, material well-being , intimacy, social status, and safety. Burnout is associated with a statistically significant decrease in productivity in the domain of objective quality of life, indicating the necessary involvement of the employers and social community in undertaking prevention intervention measures. There are many studies by other authors that obtained similar results in researching the interrelations of the phenomena that we also observed[14,15,16,17,18,19,20], and that emphasize in conclusion the necessity to apply comprehensive measures in order to prevent undesirable consequences.

**TABLE 4**
**MANOVA Significance Tests for Effects of Personal and Work Burnout, Age, Sex, and All Domains of Objective and Subjective Quality of Life**

| Variables and Types of Effects | F | p |
|---|---|---|
| All domains of objective quality of life | | |
| Effects of interactions | | |
| *Personal burnout, work burnout, age, sex* | 22.42 | * |
| *Personal burnout, work burnout, age* | 39.88 | * |
| *Personal burnout, work burnout, sex* | 45.99 | * |
| *Personal burnout, work burnout* | 16.76 | * |
| Individual effects | | |
| *Personal burnout* | 32.03 | * |
| *Work burnout* | 12.79 | * |
| *Age* | 31.55 | * |
| *Sex* | 24.51 | * |
| All domains of subjective quality of life | | |
| Effects of interaction | | |
| *Personal burnout, work burnout, age, sex* | 18.94 | * |
| *Personal burnout, work burnout, age* | 12.47 | * |
| *Personal burnout, work burnout, sex* | 7.72 | * |
| *Personal burnout, work burnout* | 19.69 | * |
| Individual effects | | |
| *Personal burnout* | 42.62 | * |
| *Work burnout* | 8.94 | * |
| *Age* | 4.45 | * |
| Sex | 19.17 | * |

*$p$ < 0.001.

**TABLE 5**
**Regression Analysis of Impact of Burnout, Quality of Life, Age, and Sex on Work Ability Index**

| Factor | Univariate Analysis | | | | | | Multivariate Analysis | | | | | |
|---|---|---|---|---|---|---|---|---|---|---|---|---|
| | B | Standardized B | R Square | 95% CI for B | | p | B | Standardized B | R Square | 95% CI for B | | p |
| | | | | Lower Bound | Upper Bound | | | | | Lower Bound | Upper Bound | |
| Personal burnout | -0.041 | -0.187 | 0.035 | -0.061 | -0.022 | * | -0.021 | -0.097 | 0.154 | -0.052 | 0.010 | — |
| Work burnout | -0.029 | -0.121 | 0.015 | -0.052 | -0.008 | ** | 0.010 | 0.042 | | -0.022 | 0.042 | — |
| Objective quality of life | 0.224 | 0.364 | 0.132 | 0.173 | 0.276 | * | 0.125 | 0.204 | | 0.054 | 0.195 | * |
| Subjective quality of life | 0.049 | 0.336 | 0.113 | 0.037 | 0.062 | * | 0.027 | 0.189 | | 0.012 | 0.043 | * |
| Age | -0.164 | -0.034 | 0.001 | -0.581 | 0.253 | — | -0.202 | -0.044 | | -0.631 | 0.228 | — |
| Female sex | -1.048 | -0.120 | 0.014 | -1.837 | -0.259 | ** | -0.909 | -0.104 | | -1.679 | -0140 | *** |

*****$p$ < 0.001, **$p$ < 0.01, ***$p$ < 0.05.

There are not many available studies with a workers' population as the subject of the mentioned phenomena, which our study observed. In a study dealing with the examination of food manufacturing workers, during an 11-year observation, Salonen et al. provide the factors associated with their early retirement as chronic diseases, long sickness leaves, work-related stress, low working and physical index, and hard physical work[21]. The results of the investigations by the authors who studied connections between illnesses, psychosocial stress at work, and work ability also emphasized the significant impact of individual personal characteristics of the workers. Personality type A, adjustment style, age, sex, education level, marital status, self-confidence, and others are the main determinants[22]. The multivariate regression analyses in our study point out objective and subjective quality of life, as well as female sex as factors significantly associated with work ability index.

A recent study that investigated the impact of individual and work factors on work ability of the exposed people, by the analysis of 14 cross-sectional and six longitudinal studies on this subject, points out unfavorable factors, such as lack of free time; obesity; high mental and physical work demands; lack of autonomous decision making; and individual sex characteristics, lifestyle, and physical conditions of the workers[23]. The authors that were interested in specific etiological factors in female sex point out the significant impact of a greater psychophysical effort, since women have two jobs – one at home and one at work[24], which is quite common in Serbia. Research studies on the connection between work and home life have found that burnout has a negative spillover effect. Workers experiencing burnout were rated by their spouses in more negative ways, and they themselves reported that their work has a negative impact on their family and that their marriage is unsatisfactory[14].

Occupational stress occurs when job demands do not match the person's adaptive resources. Stress is a generic term that refers to the temporary adaptation process that is accompanied by mental and physical symptoms. In contrast, burnout can be considered as a final stage in a breakdown in adaptation that results from the long-term imbalance of demands and resources, thus from prolonged job stress[4,15]. Burnout is a multidimensional, work-related, and primarily mental syndrome, whereas chronic fatigue syndrome is generic and predominantly characterized by unexplained fatigue and additional physical symptoms. Burnout has been associated with various forms of negative responses to the job, including job dissatisfaction, low organizational commitment, absenteeism, intention to leave the job, and turnover. People who are experiencing burnout can have a negative impact on their colleagues, both by causing greater personal conflict and by disrupting job tasks. Thus, burnout can be contagious and perpetuate itself through informal interactions on the job[1,4,15].

From the very beginning of burnout investigations (1970), scientists were interested in possible prevention of this phenomenon. It is generally accepted that the measures in stress prevention have a good effect in burnout prevention as well, and fall under two categories: person-centered and workplace-centered interventions. Meta-analyses of 48 experimental studies that dealt with analysis of prevention measure efficacy of both types of strategies indicate higher efficacy of individual, person-centered interventions[25]. However, there are opposite experiences. Boer et al. conducted a study on manufacturing workers that underwent a 6-month health-promotion program in the workplace. The group of exposed workers achieved better quality of life, higher work ability index, and lower burnout score in comparison to the control group[26]. However, a strictly individual approach to burnout creates the danger that a "blame the victim" situation is created.

## IMPLICATIONS OF THE STUDY

According to other authors' experiences concerning detailed analyses of advantages and disadvantages of intervention measures in burnout prevention, and taking into consideration the results of our study where burnout is accompanied by an excellent work ability index, we directed our interest to the salutogenic approach in burnout prevention, by health-promotion programs in the workplace that have been advocated worldwide in recent years. The ultimate objective of health-promotion activities is to create prerequisites for a good life. According to the Ottawa Charter, health promotion enables people to take control of the determinants of health in order to achieve their fullest potential[27,28,29,30,31]. The American-Israeli medical sociologist Aaron Antonovsky introduced his salutogenic theory "sense of coherence" as a global orientation to view the world, claiming that the way people view their life has a positive influence on their health. Sense of coherence explains why people in stressful situations stay well and are even able to improve their health. The origin of salutogenesis derived from interviews of Israeli women who experienced the concentration camps of the Second World War, yet stayed healthy in spite of it[32].

In health promotion, health is seen as a human right. The focus is on the coordination of activities between professions and professionals in societies. This is a positive concept, emphasizing social and personal resources as well as physical capacities. The responsibility of health-promotion action extends far beyond the health sector and health behavior to well-being and quality of life. It is a humanistic approach, having the human being and human rights at the focus again. The individual becomes an active and participating subject. The task for the professionals is to support and provide options, enabling people to make sound choices, point out the key determinants of health, to make people aware of them and able to use them. Health education is here replaced by learning about health, referring to the reciprocity of a health dialogue. The salutogenic perspective can be applied in all these stages. The salutogenic framework can create a fusion of the complexity of health and quality of life development[32,33]. The salutogenic approach in health promotion is a universal concept focusing on resources and capacities that generate health, rather than putting focus on a disease. It refers to both sexes, all ethnic groups, social classes, and cultures[34]. This approach, derived from the salutogenic theory on health, combines the societal and the individual perspective on health; includes physical, mental, social, and spiritual health; and considers people in their social and cultural context. Further, it takes into account the material and economic resources, integrates social capital, and, finally, includes ethics and human rights.

In our study, focus should be on measures to support maintenance of a high work ability index, improve it in others, and, at the same time, it would have an impact on better quality of life and burnout elimination, since these phenomena are interdependent, as our study showed. Burnout seems to be a chronic rather than a transient condition[12,13]. It appears that the balance between investments and outcomes is crucial for the development of burnout. It looks like this mechanism is working in similar ways at the interpersonal level of caregiver and recipients, and at the organizational level of employee and organization[15,24].

Here, the organization of society becomes important. The optimal society regards people as active participating subjects (society supporting human rights). Aspects of health are included in all policies. This again serves as prerequisites for a good life. Ultimately, peoples' ability to enjoy a high quality of life depends on how well society, through coherent interdisciplinary and intersector action, is able to support the process of health through the course of life. In all, such a development may create a salutogenic society[35]. This could be reached by creating environments and societies characterized of clear structures and empowering environments where people see themselves as active participating subjects who are able to identify their internal and external resources, use and reuse them to realize aspirations, to satisfy needs, to perceive meaningfulness, and to change or cope with the environment in a health-promoting manner[36]. Analysis and reviews of the studies that investigated efficacy of the salutogenic approach in health promotion in the workplace indicate its advantage in comparison to other well-known prevention approaches[37,38,39,40,41].

We hope that the results of this study will be argumentative enough for burnout to be included in the disease list and to get its code in the national disease classification, as is the case in Sweden[42]. The results of the study are also argumentative for certain changes of the law on safety in the workplace, primarily that health promotion in the workplace must be obligatory for employers and must be financed by the state.

## CONCLUSION

Burnout, quality of life, and work ability are significantly interrelated categories in food manufacturing workers. There is a high degree of work burnout that has not yet been accompanied with significant impairment of quality of living and work ability in exposed workers. That is why the salutogenic approach in prevention of this phenomenon, by health-promotion programs in the workplace, would be the method of choice in burnout improvement. Such an approach requires tripartite realization; except the workers, it is necessary that their employers and the society as a whole get engaged. Legislatives issued by the state can have a crucial role. Standardized questionnaires for burnout (CBI), quality of life (ComQol), and work ability index (WAI) were translated and they are suitable for the Serbian area, and can be used for research of this phenomenon in workers of other professions as well, and are readily available to other researchers from the same speech area.

The results of the study were limited by the short period of the examination and relatively small number of observed workers, but we hope that our already-started detailed examinations with longer trial period will result in more precise evidence.

The authors of the study believe that availability of the instruments in the Serbian language for monitoring stress consequences in Serbia could be useful for future investigations and for other researchers in the country. We also expect that the discussion will open new topics and initiate future multicentric approaches.

## ACKNOWLEDGMENTS

This study was published thanks to the Ministry of Science and Technological Development of the Republic of Serbia (Project no. 21016: Introduction and development of the coherent engagement of all economic branches and their employees in promotion of health, ethics and quality of living as a condition for their development).
